# Akinetic rigid symptoms are associated with decline in a cortical motor network in Parkinson’s disease

**DOI:** 10.1038/s41531-020-00120-3

**Published:** 2020-08-24

**Authors:** Sarah J. Kann, Chiapei Chang, Peter Manza, Hoi-Chung Leung

**Affiliations:** 1 Department of Psychology, Integrative Neuroscience Program, Stony Brook University, Stony Brook, NY USA; 2 Renaissance School of Medicine, Stony Brook University, Stony Brook, NY USA; 3 National Institute on Alcoholism and Alcohol Abuse, National Institute of Health, Bethesda, MD USA

**Keywords:** Parkinson's disease, Basal ganglia

## Abstract

The akinetic/rigid (AR) motor subtype of Parkinson’s Disease is associated with increased rates of motor and cognitive decline. Cross-sectional studies examining the neural correlates of AR have found abnormalities in both subcortical and cortical networks involved in motor planning and execution relative to controls. To better understand how these cross-sectional findings are implicated in the unique decline associated with the AR subtype, we examined whether baseline AR symptoms are associated with longitudinal decline of these networks, in contrast to other motor symptoms such as tremor. Using whole brain multiple regression analyses we found that worse AR symptoms at baseline were associated with greater gray matter loss over four years in superior parietal and paracentral lobules and motor cortex. These regions also showed altered connectivity patterns with posterior parietal, premotor, pre-supplementary motor area and dorsolateral prefrontal regions in association with AR symptoms across subjects. Thus, AR symptoms are related to gray matter decline and aberrant functional connectivity in a network of frontal-parietal regions critical for motor planning and execution. These structural and functional abnormalities may therefore be implicated in the more aggressive course of decline associated with the AR relative to tremor-dominant subtype.

## Introduction

Motor symptoms in Parkinson’s Disease (PD) are heterogenous, with the akinetic/rigid (AR) motor subtype being associated with poorer prognosis and increased risk of dementia compared to the tremor subtype^1–3^. Differential patterns of neuropathology and rates of decline across individuals with PD may therefore manifest as specific symptom profiles^4–6^. While recent cross-sectional functional and anatomical neuroimaging studies have found distinct correlates of AR symptoms, including motor and posterior parietal cortical regions as well as basal ganglia (see ref. ^7^ for review), the pattern of longitudinal changes involving these neural substrates and their association with initial AR symptoms remains largely unknown. The current study therefore examined longitudinal changes in gray matter regions associated with AR symptoms measured at a baseline time point. We also determined if such regions exhibit altered functional connectivity patterns in relation to AR symptoms, which may identify a link between early AR symptom severity and neuronal pathways associated with accelerated spread of PD pathology^6,8^.

AR symptoms are commonly defined as a composite of motor deficits classified as rigidity, bradykinesia and akinesia, while tremor symptoms are defined via items associated with postural and kinetic rhythmic involuntary movements as measured in the Movement Disorder Society Unified Parkinson’s Disease Rating Scale^9^. While both symptom types have been associated with alterations in motor cortex and basal ganglia^10–12^, critical distinctions between the two have also been proposed. Specifically, tremor symptoms have been linked to altered interactions between globus pallidus and the cerebellothalamic circuit^11,13,14^, while AR symptoms have been associated with altered anatomy and function of basal-ganglia motor loops, specifically in projections from putamen to globus pallidus, thalamus and ultimately motor cortex^15–17^. These overlapping yet distinct network alterations may offer insights into the unique longitudinal trajectory associated with each symptom dimension. Specifically, recent work has proposed that the initial decline of basal ganglia causes compensatory mechanisms within the cerebellothalamic network, resulting in tremor symptoms and possibly a more benign form of disease progression^18–20^. In contrast, neural decline associated with AR symptoms may reflect pathways of initial PD progression, thus highlighting the importance of evaluating longitudinal neural patterns associated with early AR symptoms.

While cross-sectional studies have consistently found a relationship between AR symptoms and reduced levels of putamen dopamine, activation and volume^21–24^, it has also been suggested that AR symptoms may not be entirely accounted for by dopamine depletion and its effects on basal ganglia-cortical interactions (see ref. ^15^ for review). In particular, AR symptoms have been linked to abnormalities in motor planning^7,25,26^, which is reliant on a frontal-parietal network, consisting of posterior parietal cortex, pre-supplementary motor area (SMA) and premotor cortex, which projects to motor cortex (for review see refs. ^27,28^). Several neuroimaging studies have reported associations between AR symptoms and alterations in connectivity^29,30^, activation, and volume^30–33^ of regions within this fronto-parietal network including pre-SMA, motor cortex, putamen, and parietal cortex. Overall, these findings suggest that the putamen, as well as frontal and parietal inputs to motor cortex may be closely related to AR symptom severity at discrete time points. However, to the best of our knowledge no studies have evaluated the longitudinal neural correlates of AR. This information would be critical for understanding the mechanisms by which individuals with greater AR symptoms are at increased risk for poorer outcome (e.g., faster decline and dementia).

Therefore, we sought to examine the relationship between longitudinal gray matter changes and early measures of AR symptoms in contrast to tremor symptoms. Given the literature relating AR symptoms to altered anatomy and function of two distinct circuits with inputs to the motor cortex, specifically the putamen^21,23,24^ and a frontal-parietal cortical network^30–33^, we hypothesized that these regions would show increased gray matter decline for those with more severe AR symptoms at a baseline time point. We also examined whether regions that decline anatomically at a greater rate in association with early AR symptoms would have altered functional connectivity patterns associated with individual differences in AR symptoms as well. Such analyses may therefore reveal pathways by which disease spread occurs at a greater rate for patients with more severe AR symptoms.

## Results

We first confirmed that motor symptoms progressed over the four-year period in a subset of the PPMI sample by conducting a repeated measures analysis of variance on the 274 participants who had clinical and behavioral assessments available at all four time points (See Table 1, right column). As expected, motor symptoms for both AR (*F* (3819) = 70.21, *p* < 0.001) and tremor (*F* (3819) = 14.41 *p* < 0.001) domains increased significantly over time.

**Table 1:** Average and standard deviation of variables of interest for all participants included in the resting-state functional connectivity, gray matter and behavioral analyses.

	Resting state (*N* = 87)	Gray matter (*N* = 50)	Behavior (*N* = 274)
Age	61.14 (10.22)	59.62 (9.66)	60.26 (9.85)
Gender (female)	29	16	85
Years of education	15.27 (2.9)	14.36 (3.29)	15.5 (2.87)
H & Y Stage	1.7 (.53)	1.40 (.49)	1.51 (.51)
UPDRS III (total)	17.68 (9.22)	17.78 (8.55)	18.95 (8.47)
AR subscale	9.73 (5.41)	9.72 (4.83)	10.20 (5.50)
Tremor subscale	2.39 (2.50)	2.54 (2.15)	2.90 (2.15)
LED at scan	188.53 (211.13)	544 (260)	NA

Gray matter and behavioral data were analyzed across four time points and have baseline demographics listed above, whereas resting fMRI data are from a single time point and have demographics from time of scan listed above.

*H&Y* Hoehn and Yahr, *UPDRS* unified Parkinson’s progressive marker initiative, *AR* akinetic/rigidity, *LED* levodopa dose equivalent.

### Baseline measures of AR and longitudinal changes in gray matter

**Fig. 1:**
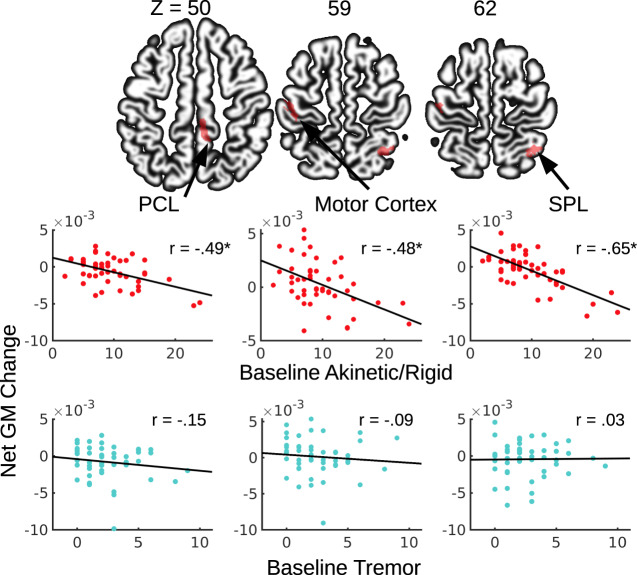
Motor symptoms and gray matter volume change over time. Greater gray matter volume loss over 4 years was associated with AR but not tremor symptom severity at baseline. SPL superior parietal lobule, PCL paracentral lobule.

To examine the extent to which baseline motor symptoms related to basal ganglia and cortical gray matter values over time, we estimated the impact of baseline AR and tremor symptom severity on net gray matter changes across the four time points (baseline, 12, 24, and 48 months) by applying multiple regression analyses on the voxel compression maps (VCM) while controlling for confounding factors (see “Methods”). In a sub-sample of 50 participants, individuals with more severe AR symptoms at baseline showed significantly greater net gray matter volume loss across the 4 years in the paracentral (PCL) and posterior superior parietal lobule (SPL) at the whole brain level (Table 2). Though one participant’s rate of PCL decline was more than three standard deviations beyond the mean, the correlations between AR and gray matter decline in PCL remained significant after removing this outlier (Fig. 1) and the strength of the correlation did not change significantly (*z* = −0.47, *p* = 0.63). Additionally, a cluster in the motor cortex was prominent, though not meeting the cluster corrected significance threshold (*k* = 148, *p* = 0.11) (Table 2). After removing one outlier, the relationship between AR and gray matter decline in the motor cortex remained significant (Fig. 1) and the strength of the correlation did not change significantly (*z* = −0.46, *p* = 0.64). Surprisingly, no significant relationship between AR scores and putamen volume decline were found, even at a more lenient threshold (*p* < 0.005).

**Table 2:** Gray matter clusters from change maps that are associated with baseline AR scores.

Cluster size	*z* score	*x*	*y*	*z*	Identified region
217	4.62	−1	−67	58	PCL
532	5.04	35	−56	63	SPL
148	4.24	−44	−19	59	Motor Cortex

Motor cortex failed to meet cluster corrected significance.

*SPL* superior parietal lobule, *PCL* paracentral lobule.

In contrast, baseline tremor scores did not show any significant relationship with longitudinal gray matter volume changes in the whole brain analyses. The correlations between tremor and gray matter loss in ROIs defined in the above AR analysis were also not statistically significant (Fig. 1). Critically, we found that the correlations between gray matter decline in the above ROIs and AR symptoms were significantly different from the correlations between decline of these ROIs and tremor symptoms (*z*’s = −1.96 − −4.215, *p*’s = <0.001–0.04)^34,35^.

### Measures of AR and functional connectivity across subjects

We further utilized multiple regression analyses to examine the across-subject relationship between motor symptoms and functional connectivity using a sub-sample of 87 participants with resting-state fMRI data. We specifically examined whether the regions with significant longitudinal gray matter loss would also show alterations in their functional connectivity in association with higher AR symptoms.

**Fig. 2:**
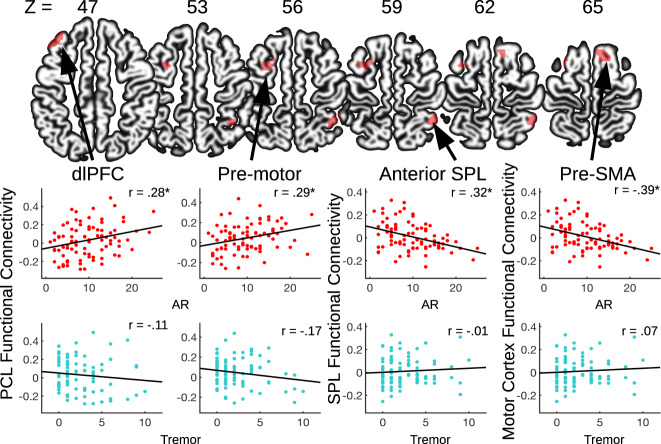
Motor symptoms and resting-state functional connectivity. Resting-state functional connectivity maps showed altered cortical connectivity in association with AR symptom severity at time of scan. The three seed regions are shown in Fig. 1. SPL superior parietal lobule, PCL paracentral lobule, dIPFC dorsolateral prefrontal cortex.

Individual variations in AR symptoms were significantly associated with posterior superior parietal lobule (SPL) functional connectivity; this parietal region showed weaker functional connectivity with the anterior portion of the SPL in relation to more severe AR symptoms (see Fig. 2 and Table 3). Greater AR symptom severity was also associated with weaker functional connectivity between motor and pre-SMA. (see Fig. 2 and Table 3). Individual variation in AR symptoms were not strongly associated with paracentral lobule (PCL) functional connectivity at the *p* < 0.001 voxel-wise threshold, though at a reduced threshold (*p* < 0.005), there was an association between AR symptoms and stronger functional connectivity between PCL and the left premotor cortex and dorsolateral prefrontal cortex (dlPFC) (see Fig. 2 and Table 3). These across-subject correlations between motor symptoms and functional connectivity were not significantly different between the 36 participants included in both anatomical and connectivity analyses and the 51 participants only included in the connectivity analysis (*z*’s = 0.01 – 0.38, *p*’s = 0.70 – 0.99), except for the dorsolateral prefrontal cluster which trended towards significance (*z* = 1.99, *p* = 0.05). However, the actual resting-state values extracted from this cluster did not significantly differ between the groups *(t* = −0.134, *p* = 0.89). Additionally, these functional connectivity patterns were not significantly associated with tremor symptom severity (see Fig. 2), while the correlations between functional connectivity in the above ROIs and AR symptoms were significantly different from the correlations between functional connectivity and tremor symptoms (*z*’s = 1.78 – 2.48, *p*’s = 0.01 – 0.07)^34,35^.

**Table 3:** Clusters from resting-state functional connectivity maps associated with AR symptoms.

Resting-state functional connectivity Seed	Cluster size	*z* score	*x*	*y*	*z*	Identified region
Posterior SPL	41	4.40	42	−46	59	anterior SPL
Motor Cortex	40	4.77	9	20	62	pre-SMA
		Reduced threshold *p* < 0.005				
PCL	41	4.26	−27	5	56	premotor
PCL	39	4.39	−36	35	47	dlPFC

PCL map is presented at a lower vowel-wise threshold.

*SPL* superior parietal lobule, *pre-SMA* pre-supplementary motor area*, PCL* paracentral lobule, *dIPFC* dorsolateral prefrontal cortex.

## Discussion

The current study demonstrated that PD individuals with more severe AR symptoms exhibited greater gray matter volume reduction over four years and altered functional connectivity within a frontal-parietal network involved in motor planning and execution. Critically, these patterns of gray matter decline and altered functional connectivity were not found for the tremor subscores. The implications of these findings are discussed below.

Our findings support our initial hypothesis that baseline AR symptoms related to volumetric changes and altered functional connectivity within a frontal-parietal network^27,36^. Specifically, we found longitudinal anatomical decline associated with baseline AR symptoms in superior parietal lobule (SPL), a region heavily implicated in motor planning and awareness, as well as paracentral lobule (PCL) and motor cortex, regions primarily involved in the execution of planned movements^37–40^. These findings corroborate studies that have found lower gray matter volume and altered activation in SPL and cortical motor areas in relation to AR symptoms^31,33^. Similar patterns of reduced gray matter in the SPL, PCL and motor areas have also been found in several studies that compare PD patients and healthy controls^41,42^. However, these latter studies did not distinguish between AR and tremor subtypes, suggesting that patterns of anatomical changes associated with PD categorically may well be related to distinct motor profiles within the disease. Our findings therefore further implicate frontal and parietal motor control regions in the unique longitudinal trajectory specifically associated with AR symptoms.

We also found altered functional connectivity of the SPL, PCL, and motor cortex in relation to AR symptoms, which is in line with several studies that have found functional alterations in motor cortex and parietal regions in relation to AR symptoms^7,29,30^. Specifically, we found weaker connectivity between anterior and posterior portions of SPL in relation to worse AR symptoms. Functional parcellations of the SPL have found the anterior segments to be more strongly connected with sensory processing regions, while posterior segments are more strongly connected with regions involved in executive functions^43^. Accordingly, with the current findings, it is possible that AR symptoms are related to abnormal integration of these sensory and motor planning processes that occur within the SPL. Additionally, we found altered connectivity in relation to AR symptoms between regions responsible for motor output (motor cortex and PCL) and regions involved in motor planning, including premotor cortex, dorsolateral prefrontal cortex, and pre-SMA^44–46^. Similar to the altered SPL connectivity, altered connectivity between these motor regions in relation to AR suggests altered integration of motor planning with execution, though studies evaluating this with direct experimentation are needed to substantiate this claim.

Furthermore, several of the regions which exhibited altered connectivity in relation to AR are also implicated in cognitive control processes. In particular, the connectivity between pre-SMA and motor cortex has been found to play a central role in response inhibition^47^, while activity of dorsolateral prefrontal cortex has been strongly implicated in working memory tasks and visuospatial information processing^48–50^. Additionally, impairments in inhibition and working memory in PD patients have been associated with alterations in these regions^51–53^, while abnormalities in these cognitive control systems may also play a role in mood disturbances in PD^54–56^. Our findings are therefore in line with previous studies that have found a strong relationship between AR symptoms and executive function impairments in PD patients (see ref. ^57^ for review), symptoms which may have common underlying neural correlates. Future studies should directly evaluate this possibility using longitudinal methods in order to define neural decline that may relate to both cognitive and motor domains.

Finally, as both cortical and subcortical networks input to the motor cortex, a region we found to be associated with AR symptoms, we cannot rule out the relationship between decline of subcortical systems and initial AR symptoms. However, as we did not find significant AR-related anatomical decline in the putamen, a region that was expected to be directly affected by the progressive death of dopamine neurons associated with PD pathology^58–60^, our results do highlight the potential role of networks beyond the basal-ganglia-cortical loop in AR symptoms. This is in line with a growing body of literature that has begun to examine the relationship of PD symptoms, such as tremor and memory decline, and dysfunction of systems beyond dopaminergic basal ganglia loops^13,61–63^. Future studies interested in differentiating the neural correlates of motor symptoms in PD, as well as how these neural correlates decline over time, should therefore expand their analyses to include systems beyond those associated with basal ganglia function, specifically frontal and parietal regions involved in motor planning and output.

Additionally, our work is the first, to the best of our knowledge, to demonstrate that AR symptoms have distinct patterns of anatomical decline that are associated with altered functional connectivity, in contrast to tremor symptoms. This relationship is critical as recent work in PD has substantiated the hypothesis that neurodegenerative disease pathology spreads along the brain’s connectome^8,64^, with studies finding that functional network paths predict regions of increased anatomical decline in PD patients^6^. While we did not directly test the disease spread hypothesis in our current work, our findings lend support for this hypothesis, and further suggest that AR symptoms are associated with distinct patterns of neural decline and disease spread along cortical networks integrating motor planning with execution. Additionally, future work aiming to evaluate disease spread in PD should take motor symptom subcategories into consideration. Evaluating disease spread via AR and tremor subtypes may ultimately provide critical information for disease prognosis and treatment planning, with future work ideally utilizing fully overlapping participants in both anatomical and functional analyses.

Our study had several limitations that should be noted. Although the resting-state analysis was informed by the longitudinal gray matter findings, not all participants had data of both neuroimaging modalities available for analysis. While the differences in sample is an important limitation, there were no significant differences in the demographic variables or the extracted resting-state values between the group of participants included in both neuroimaging analyses and those included only in the resting-state analysis. Future studies though should ideally integrate multiple modalities in completely overlapping samples. Additionally, larger sample sizes would afford better detection of subtle longitudinal changes in structural and functional images. Our analysis did demonstrate that individual differences in baseline AR symptom severity related to gray matter decline in frontal and parietal regions involved in motor planning and execution, while functional connectivity in motor planning regions was altered in relation to AR symptoms as well. These regions therefore may play a critical role in the unique longitudinal trajectory taken by AR dominant PD patients. Such findings may inform future studies aiming to understand the longitudinal trajectory of AR dominant PD patients, and ultimately help in disease prognosis and planning for such patients.

## Methods

### Participants

Behavioral and imaging data were obtained from the Parkinson’s Progression Markers Initiative (PPMI) database (www.ppmi-info.org/data). PPMI—a public-private partnership—is funded by the Michael J. Fox Foundation for Parkinson’s Research and partners, including Abbvie, Avid Radiopharmaceuticals, Biogen, Bristol-Meyers Squibb, Covance, GE Healthcare, Genetech, GlaxoSmithKline, Eli Lilly, Lundbeck, Merck, Meso Scale Discovery, Pfizer, Piramal, Roche, and UCB. This multi-site study collected standardized behavioral, neuropsychological, biospecimen and neuroimaging data from a group of 424 newly diagnosed individuals with PD, and follow-up data up to four years (12, 24, and 48 months) after initial assessments. More specific details of the PPMI study can be found in a report published previously^65^. For up-to-date information on the study, visit the project webpage (www.ppmi-info.org). All participants enrolled in the PPMI study within 24 months of their initial diagnosis. Each participating PPMI site received approval from an ethical standards committee on human experimentation before study initiation. Written informed consent for research was obtained from all individuals participating in the study.

### Behavioral and clinical data

Each patient’s overall motor impairment was indexed using the total (summed) score of the Unified Parkinson’s Disease Rating Scale (UPDRS) part III^9^, where higher scores indicate more severe overall motor symptoms. While some previous studies have examined motor subtypes by classifying patients into AR or tremor-dominant groups^66^, we chose to examine individual differences in these two domains of motor symptoms. Each individual’s AR score was calculated as a sum of UPDRS III ratings on rigidity, finger tapping, hand movements, arising from chair, posture, gait, and body bradykinesia, and the tremor scores as a sum of ratings on resting tremor of the arms and legs and action tremor of the arms (see ref. ^67^). To determine if motor impairments became more severe over time we evaluated motor domain scores in 274 patients who had behavioral data collected at all four time points. Baseline demographic information regarding these participants can be found in Table 1.

### Imaging data

Imaging data were downloaded to local computers in January 2019. Structural images were available at all four time points from 87 participants, though only 50 participants had scanning parameters that were consistent and reflected those provided in the PPMI documentation (see below). We therefore used the scans of these 50 individuals (16 female, 47 on PD medication) in the final longitudinal volumetric analysis, 47 of whom also were included in the longitudinal behavioral analysis. It is worthy to note that these 47 participants had comparable baseline demographics relative to the 227 behavioral participants not included in the anatomical analysis (*t*’s = 0.144 – 1.39, *p*’s = 0.16 − 0.88). The imaging data were acquired at seven different sites (Baylor College of Medicine, Johns Hopkins, Emory, Northwestern, Tübingen, Marburg, and Cleveland Clinic). All imaging (MRI and fMRI) data were acquired using Siemens Trio Tim 3 Tesla magnets (Siemens Medical Systems, Erlangen, Germany). We only included individuals who were not reported by the original experimenters as having any problems during the scanning session (e.g., experience of claustrophobia). Individual participants’ anatomical and functional images were visually inspected to ensure that the whole brain was covered and were screened for artifacts. Data were obtained from participants with the imaging parameters specified in the PPMI documentation as the following: high resolution structural images (T1-weighted MP-RAGE) were acquired with repetition time (TR) = 2,300 ms, echo time (TE) = 2.98 ms, flip angle (FA) = 9°, matrix = 256 × 256, field of view (FOV) = 256 mm, voxel size 1 × 1 × 1.2 mm^3^, 176 sagittal slices with slice thickness = 1.2 mm. Subjects were instructed to rest quietly, to keep their eyes open and not to fall asleep. Anatomical scans were visually inspected for all time points, and no participants were removed due to motion or other artifacts. At the 48-month time point the mean levodopa equivalent dose (LED)^68^ was 544 mg/day (standard deviation = 260 mg/day) (see Table 1).

Resting-state functional images were acquired from the baseline, 12, 24, and 48-month time points, which included 18, 55, 64, and 78 scans respectively, though only 62 and 55 scans out of the total 24 and 48-month time points had consistent scanning parameters as defined in the PPMI documentation (see below). As the goal of our study was to determine if anatomical regions defined in our longitudinal gray matter analysis had functional implications, we chose to select the first time point at which functional scans were available for each participant, so as to obtain the highest number of functional scans possible. We therefore analyzed 94 participants with resting-state scans from the first time point at which each individual was scanned with consistent scanning parameters. Data were obtained with the imaging parameters specified in the PPMI documentation as following: Echo-planar images acquired for 8.29 min (212 volumes) with TR = 2400 ms, TE = 25 ms, flip angle = 80°, matrix = 68 × 68, FOV = 222 ×222 mm, 40 slices (ascending with 0-mm gap), and voxel size 3.25 × 3.25 × 3.25 mm^3^. Data from five participants were excluded from analysis because of severe artifacts, and additionally data from two participants were excluded due to excessive head motion during fMRI (for details, see Head Motion section below). As a result, a total of 87 individuals (29 female) were included in the final resting-state functional connectivity analysis, 36 of whom had sufficient anatomical scans available to be included in the longitudinal anatomical analysis. Critically, these 36 subjects had motor and demographic characteristics comparable to the 51 subjects that only had resting-state scans analyzed (*t*’s = −1.2 – 1.2, *p*’s = 0.11 – 0.74), thus allowing for informative interpretation to be made of results across the neuroimaging data types. The mean LED at time of scan was 188 mg/day (standard deviation = 211 mg/day). The participants on medication did not undergo a medication washout before scanning. Table 1 shows the demographic information of these participants included in the final analysis.

### Longitudinal gray matter volume analysis

Structural MRI data were processed using the computational anatomy toolbox (CAT12 r930) (http://dbm.neuro.uni-jena.de/cat12/) and SPM 12. For each participant, T1 images from each of the four time points were first registered and bias-corrected using serial longitudinal registration in SPM 12^69^. In this process each time point is reoriented, spatially warped, and intensity biased corrected relative to the average T1 image, resulting in four jacobian difference maps representing the extent to which voxels from each time point image expand (values > 1) or compress (values < 1) in relation to the average T1 image. Individuals’ average T1 images were then segmented into gray and white matter and cerebral spinal fluid using CAT12.

Net gray matter change was computed in a fashion similar to that implemented in recent studies (e.g., ref. ^70^). More specifically, for each subject, we constructed a within-subject linear model in which each jacobian determinant image was regressed against time in years. The resulting beta image was then multiplied by the gray matter segmentation of the average T1 image for each subject. The product of this multiplication thus represented the net change of gray matter from baseline to 48 months, and is known as a voxel compressions map (VCM)^70^. Additionally, the within-subject average gray and white matter segmentation images were used to create a group specific DARTEL template^71^. Deformation fields from this process related each subject’s average gray matter segmentation to the DARTEL template and were used to normalize VCMs to the DARTEL template. Normalized VCMs were then smoothed by 4-mm FWHM.

A group level multiple regression model was conducted in which baseline indexes for AR and tremor symptoms were used to predict gray matter changes (VCM maps), while age, LED at the final time point, scanner site, gender, and total intracranial volume at baseline were included in these models to control for confounding factors. Previous studies have shown that the corrected voxel peak threshold of *p* < 0.05, based on the Gaussian random field theory, may be too restrictive, and suggested the use of a cluster threshold^72,73^. Unless otherwise noted, we present results that satisfy both an uncorrected threshold of *p* < 0.001 at the voxel level and a false discovery rate (FDR) of *p* < 0.05 at the cluster level to correct for Type I error^74^.

### Functional image data preprocessing

Standard image preprocessing was performed using Statistical Parametric Mapping (SPM12, www.fil.ion.ucl.ac.uk/spm). For each individual, functional images were corrected for slice timing and then realigned to the first image to correct for head motion between scans, while structural image was coregistered to the mean functional image. The structural image was then segmented and normalized to a template based on a sample of individuals with PD^75^ using affine registration followed by nonlinear transformation^76,77^. The resulting parameters were then applied to all functional images. Finally, the functional images were spatially smoothed with a Gaussian kernel of 6 mm at full width at half maximum.

Additional preprocessing was applied to reduce spurious fMRI signal variances that were unlikely to reflect neuronal activity^78–81^. We quantified spurious variance using the aCompCor method^82^, which applies a principal components analysis to extract signal from the ventricles and white matter, and includes these nuisance signals and their first and second order derivatives as regressors in the final time series. We chose not to include global signal regression, as recent work^83^ has demonstrated that the aCompCor method is able to produce resting-state time series comparable to methods which include global signal regression. Scrubbing and censoring were conducted on the resulting time series using frame displacement (FD) and derivative of the root mean square variance over voxels (DVARS) as in our previous work^84–87^. Each functional volume that exceeded the head motion limit FD (*t*) > 0.5 mm or DVARS(*t*) > 0.5% were included as a regressor in the final time series^87,88^. Two out of the 94 (2.12 %) processed resting-state datasets had >1/3 of time points that exceeded the motion limit, while five subjects were removed due to artifacts. On average, 9.3% of data points exceeded the motion limit in the remaining 87 datasets. The number of scans exceeding motion limits did not significantly correlate with the UPDRS III total score, or the AR and tremor subscales (*r*’s = −0.08 − 0.06, *p*’s = 0.46 − 0.56).

### Seed-based resting-state functional connectivity

ROIs with significantly steeper longitudinal gray matter decline in association with higher AR scores were used as seeds in our resting-state analyses. The fMRI signal time courses were averaged across all voxels within each seed region. For each resting-state fMRI dataset, we computed the correlation coefficient between the averaged time course of each seed region and the time course of each voxel in the rest of the whole brain. The resulting r values were converted to *z* scores using the Fisher’s *z* transformation^89,90^: *z* = 0.5 log (1 + *r*) / (1 − *r*). The resulting *z* maps from each individual were used in the second-level multiple regression analyses.

The main analysis consisted of a group level multiple regression model for each seed region to determine the relationship between individual motor scores at time of scan and seed-based functional connectivity differences across subjects. These models also included the confounding factors of age, gender, scanner site, and LED at time of scan, and time point at which the resting-state scan was obtained. Unless otherwise noted, we present results that satisfy both an uncorrected threshold of *p* < 0.001 at the voxel level and a false discovery rate (FDR) of *p* < 0.05 at the cluster level to correct for Type I error, in line with current reporting guidelines^74^. A summary table for all multiple regression analyses conducted can be found in Supplementary Table 1.

### Reporting summary

Further information on research design is available in the Nature Research Reporting Summary linked to this article.

## Supplementary information

Supplemental Material

reporting summary

## Data Availability

Data used for this study were obtained from the PPMI database (www.ppmi-info.org/data). For up-to-date information on the study, visit http://www.ppmi-info.org.

## Code availability

Codes used in this study are available from the authors upon request.
