# Supplementary material for: Akinetic rigid symptoms are associated with decline in a cortical motor network in Parkinson’s disease
Source: NPJ Parkinsons Dis. 2020 Aug 24;6:19. doi: 10.1038/s41531-020-00120-3 (PMC7445297; doi:10.1038/s41531-020-00120-3)
Supplement: Supplementary file 1 — Supplemental Material [file 41531_2020_120_MOESM1_ESM.pdf]

Supplementary Table 1.

Image Analysis Types	Seed regions
Resting State	Anterior SPL
Resting State	Motor Cortex
Resting State	PCL
VCM	n/a

Supplementary Table 1. *Summary of multiple regression analyses performed, imaging analysis types, and seed regions (for resting state). Abbreviations: Superior Parietal Lobule (SPL), Paracentral Lobule (PCL), Voxel Compression Maps (VCM).*
