# Supplementary material for: Akinetic rigid symptoms are associated with decline in a cortical motor network in Parkinson’s disease
Source: NPJ Parkinsons Dis. 2020 Aug 24;6:19. doi: 10.1038/s41531-020-00120-3 (PMC7445297; doi:10.1038/s41531-020-00120-3)
Supplement: Supplementary file 2 — reporting summary [file 41531_2020_120_MOESM2_ESM.pdf]

nature research

Corresponding author(s): DBPR NPJPARKD-00401R

Last updated by author(s): Jun 26, 2020

Reporting Summary

Nature Research wishes to improve the reproducibility of the work that we publish. This form provides structure for consistency and transparency in reporting. For further information on Nature Research policies, see our Editorial Policies and the Editorial Policy Checklist.

Statistics

For all statistical analyses, confirm that the following items are present in the figure legend, table legend, main text, or Methods section.

n/a	Confirmed	
☐	☒	The exact sample size (n) for each experimental group/condition, given as a discrete number and unit of measurement
☐	☒	A statement on whether measurements were taken from distinct samples or whether the same sample was measured repeatedly
☐	☒	The statistical test(s) used AND whether they are one- or two-sided<br>*Only common tests should be described solely by name; describe more complex techniques in the Methods section.*
☐	☒	A description of all covariates tested
☐	☒	A description of any assumptions or corrections, such as tests of normality and adjustment for multiple comparisons
☐	☒	A full description of the statistical parameters including central tendency (e.g. means) or other basic estimates (e.g. regression coefficient) AND variation (e.g. standard deviation) or associated estimates of uncertainty (e.g. confidence intervals)
☐	☒	For null hypothesis testing, the test statistic (e.g. F, t, r) with confidence intervals, effect sizes, degrees of freedom and P value noted<br>*Give P values as exact values whenever suitable.*
☒	☐	For Bayesian analysis, information on the choice of priors and Markov chain Monte Carlo settings
☐	☒	For hierarchical and complex designs, identification of the appropriate level for tests and full reporting of outcomes
☐	☒	Estimates of effect sizes (e.g. Cohen's d, Pearson's r), indicating how they were calculated

Our web collection on statistics for biologists contains articles on many of the points above.

Software and code

Policy information about availability of computer code

Data collection	Data was downloaded from the Parkinson's Progressive Marker Initiative in January 2019
Data analysis	Data were processed using statistical parametric mapping (SPM) version 12, run on MATLAB 2019a

For manuscripts utilizing custom algorithms or software that are central to the research but not yet described in published literature, software must be made available to editors and reviewers. We strongly encourage code deposition in a community repository (e.g. GitHub). See the Nature Research guidelines for submitting code & software for further information.

Data

Policy information about availability of data

All manuscripts must include a data availability statement. This statement should provide the following information, where applicable:

- Accession codes, unique identifiers, or web links for publicly available datasets
- A list of figures that have associated raw data
- A description of any restrictions on data availability

The data that support the findings of this study are available from the corresponding author upon reasonable request.

April 2020

Field-specific reporting

Please select the one below that is the best fit for your research. If you are not sure, read the appropriate sections before making your selection.

☒ Life sciences ☐ Behavioural & social sciences ☐ Ecological, evolutionary & environmental sciences

For a reference copy of the document with all sections, see nature.com/documents/nr-reporting-summary-flat.pdf

Life sciences study design

All studies must disclose on these points even when the disclosure is negative.

Sample size	Sample size was determined by number of subjects available from online data base
Data exclusions	Exclusion criteria were based on motion parameters established in the neuroimaging literature
Replication	These were neuroimaging analyses that would require a separate database to replicate
Randomization	Age, gender and center site were controlled for in multiple regression analyses.
Blinding	N/A analyses of symptoms and neuroimaging

Reporting for specific materials, systems and methods

We require information from authors about some types of materials, experimental systems and methods used in many studies. Here, indicate whether each material, system or method listed is relevant to your study. If you are not sure if a list item applies to your research, read the appropriate section before selecting a response.

Materials & experimental systems

n/a	Involved in the study	
☒	☐	Antibodies
☒	☐	Eukaryotic cell lines
☒	☐	Palaeontology and archaeology
☒	☐	Animals and other organisms
☐	☒	Human research participants
☒	☐	Clinical data
☒	☐	Dual use research of concern

Methods

n/a	Involved in the study	
☒	☐	ChIP-seq
☒	☐	Flow cytometry
☐	☒	MRI-based neuroimaging

Human research participants

Policy information about studies involving human research participants

Population characteristics	Age and gender are described in the manuscript for all groups analyzed
Recruitment	Online database
Ethics oversight	Online database approved ethics

Note that full information on the approval of the study protocol must also be provided in the manuscript.

Magnetic resonance imaging

Experimental design

Design type	resting state and anatomical
Design specifications	N/A
Behavioral performance measures	N/A

Acquisition

Imaging type(s): functional and structural

Field strength: 3

Sequence & imaging parameters: (T1-weighted MP-RAGE) were acquired with repetition time (TR) = 2,300 ms, echo time (TE) = 2.98 ms, flip angle (FA) = 9°, matrix = 256 × 256, field of view (FOV) = 256 mm, voxel size 1 × 1 × 1.2 mm3, 176 sagittal slices with slice thickness = 1.2 mm. Echo-planar images acquired for 8.29 min (212 volumes) with TR = 2,400 ms, TE = 25 ms, flip angle = 80°, matrix = 68 × 68, FOV = 222 x 222 mm, 40 slices (ascending with 0-mm gap), and voxel size 3.25 × 3.25 × 3.25 mm3.

Area of acquisition: whole brain

Diffusion MRI ☐ Used ☒ Not used

Preprocessing

Preprocessing software: SPM 12 for all preprocessing

Normalization: Nonlinear normalization

Normalization template: Template derived from Parkinson's patients Xiao et al. 2017 (see manuscript)

Noise and artifact removal: We quantified spurious variance using the aCompCor method, which applies a principal components analysis to extract signal from the ventricles and white matter, and includes these nuisance signals and their first and second order derivatives as regressors in the final time series. We chose not to include global signal regression, as recent work has demonstrated that the aCompCor method is able to produce resting state time series comparable to methods which include global signal regression.

Volume censoring: Scrubbing and censoring were conducted on the resulting time series using frame displacement (FD) and derivative of the root mean square variance over voxels (DVARS)

Statistical modeling & inference

Model type and settings: The main analysis consisted of a group level multiple regression model for each seed region to determine the relationship between individual motor scores and seed-based functional connectivity differences across subjects. These models also included the confounding factors of age, gender, and LED at time of scan, and time point at which the resting state scan was obtained.

Effect(s) tested: Significance of cluster in specific contrasts of multiple regression analyses.

Specify type of analysis: ☒ Whole brain ☐ ROI-based ☐ Both

Statistic type for inference (See Eklund et al. 2016): Cluster wise, we present results that satisfy both an uncorrected threshold of $p < 0.001$ at the voxel level and a false discovery rate (FDR) of $p < .05$ at the cluster level to correct for Type I error, in line with current reporting guidelines

Correction: we present results that satisfy both an uncorrected threshold of $p < 0.001$ at the voxel level and a false discovery rate (FDR) of $p < .05$ at the cluster level to correct for Type I error, in line with current reporting guidelines

Models & analysis

n/a	Involved in the study
☐	☒ Functional and/or effective connectivity
☒	☐ Graph analysis
☒	☐ Multivariate modeling or predictive analysis

Functional and/or effective connectivity: The fMRI signal time courses were averaged across all voxels within each seed region. For each resting-state fMRI dataset, we computed the correlation coefficient between the averaged time course of each seed region and the time course of each voxel in the rest of the whole brain. The resulting r values were converted to z scores using the Fisher's z transformation : $z = 0.5 \log (1 + r) / (1 - r)$. The resulting z maps from each individual were used in the second-level multiple regression analyses.
